# Obstetric near miss and deaths in public and private hospitals in Indonesia

**DOI:** 10.1186/1471-2393-8-10

**Published:** 2008-03-12

**Authors:** Asri Adisasmita, Poppy E Deviany, Fitri Nandiaty, Cynthia Stanton, Carine Ronsmans

**Affiliations:** 1 Centre for Family Welfare, Faculty of Public Health, University of Indonesia, Depok, Indonesia; 2 Epidemiology and Population Health, London School of Hygiene and Tropical Medicine, London, UK; 3 Department of Population, Family and Reproductive Health, The Johns Hopkins Bloomberg School of Public Health, Baltimore, Maryland, USA

## Abstract

**Background:**

Falling numbers of maternal deaths have stimulated an interest in investigating cases of life threatening obstetric morbidity or near miss. The purpose of this study was to document the frequency and causes of near miss and maternal deaths in four hospitals in West Java, Indonesia.

**Methods:**

Cross sectional study in four hospitals in two districts in Banten province, Indonesia. We reviewed registers and case notes to identify the numbers and causes of near miss and death between November 2003 and October 2004. Near miss cases were defined based on organ dysfunction, clinical and management criteria. Near miss were categorized by whether or not the woman was at a critical state at admission by reviewing the final signs at admission.

**Results:**

The prevalence of near miss was much greater in public than in private hospitals (17.3% versus 4.2%, p = 0.000). Hemorrhage and hypertensive diseases were the most common diagnoses associated with near miss, and vascular dysfunction was the most common criterion of organ dysfunction. The occurrence of maternal deaths was 1.6%, with non-obstetric complications as the leading cause. The majority (70.7%) of near miss in public hospitals were in a critical state at admission but this proportion was much lower in private hospitals (31.9%).

**Conclusion:**

This is the first study to document near miss in public and private hospitals in Indonesia. Close to a fifth of admissions in public hospitals were associated with near miss; and the critical state in which the women arrived suggest important delays in reaching the hospitals. Even though the private sector takes an increasingly larger share of facility-based births in Indonesia, managing obstetric emergencies remains the domain of the public sector.

## Background

Falling numbers of maternal deaths in developed countries have stimulated an interest in investigating cases of life threatening obstetric morbidity or near miss. The advantages of near miss over death are that near miss are more common than maternal deaths, their review is likely to yield useful information on the pathways that lead to severe morbidity and death, investigating the care received may be less threatening to providers because the woman survived, and one can learn from the women themselves since they can be interviewed about the care they received [1,2].

This growing interest is reflected in an increasing number of systematic reviews on the prevalence of near miss [3-5]. The reported prevalence has ranged overall from less than 1 per 1000 live births to 82 per 1000 live births, with rates in resource poor settings ranging from four to eight percent of hospital-based deliveries [3,4]. Souza (2006) reports a mean for near miss cases of 8 per 1000 live births [5]. The variation is largely due to differences in the populations studied, but also in the definitions used. Near miss are not easy to define, and definitions have relied on a variety of approaches, including criteria of organ dysfunction; criteria of clinical management such as admission to intensive care; signs and symptoms; or clinical entities such as eclampsia or uterine rupture [3-5].

At the hospital level, an investigation into the pattern causes and timing of near miss can inform the needs for preventive programs and health care resources [6]. Hospital data on near miss can also partly inform what happens in the community however, particularly if near miss are defined at the extreme end of the severity spectrum, and are unlikely to survive if unaided by effective care in the hospital. The purpose of this study was to document the frequency, causes and timing of near miss and deaths in four hospitals in West Java, Indonesia. We report the definitions used, the frequency of near miss and death among various clinical entities, and the timing of near miss relative to the timing of admission.

## Methods

The study was conducted in four hospitals in Pandeglang and Serang districts in Banten province. Two hospitals were public hospitals (district hospitals in Serang and Pandeglang) and two were private (Budi Asih and Kencana hospitals in Serang). All hospitals perform obstetric surgery, but only the public hospital in Serang has an intensive care unit and very severe cases may be referred there. The four hospitals cover almost all hospital admissions related to pregnancy and childbirth in the two districts. In these two districts approximately only 8% of births occur in a hospital, and the findings reported here apply to hospital births only.

Near miss cases were defined as cases of life-threatening complications in women admitted during pregnancy, labor or postpartum who survived, adapting the criteria proposed by Mantel et al (1998) [6]. The latter defined near miss based on organ dysfunction, using clinical criteria related to specific disease entities as well as management criteria. Final criteria for near miss in this study were developed during workshops in Jakarta and Serang in August 2004, bringing together obstetricians, midwives and epidemiologists from Jakarta, Pandeglang, Serang and the United Kingdom. Criteria were grouped under three categories: specific organ dysfunction, general management-based criteria and specific clinical diagnoses (eclampsia, uterine rupture and ectopic pregnancy) (Table 1).

**Table 1:** Criteria for inclusion of near miss cases (modified from Mantel et al 1998)

**Organ dysfunction**	
Cardiac dysfunction	Pulmonary oedema, cardiac arrest, cardiac failure
Pulmonary embolism	-
Vascular dysfunction	Hypovolaemia requiring two or more units of blood, blood loss with hypovolaemic shock (systolic blood pressure < 90 mmHg or undetectable pulse), infusion and/or transfusion of > = 1 litre in 2 hours, free flow infusion^a^, massive haemorrhage recorded in notes
Immunological dysfunction	Septic shock
Respiratory dysfunction	Intubation or ventilation for reasons other than general anaesthesia, oxygen saturation on pulse oximetry < 90% leading to ventilation
Renal dysfunction	Oliguria < 30 ml per hour or < 400 ml per 24 hours, shock not responsive to intravascular rehydration or diuresis, haemodialysis
Liver dysfunction	Jaundice in pre-eclampsia, abnormal liver function tests
Coagulation dysfunction	Acute thrombocytopenia, prolonged bleeding time, abnormal Activated Partial Thromboplastine Time (APTT) or Prothrombine Time (PT), coagulopathy
Cerebral dysfunction	Coma, cerebral oedema, seizures other than eclampsia

**Management based criteria**	

	Intensive care admission, emergency hysterectomy, needs resuscitation, anaesthetic accident, referral to tertiary hospital

**Clinical diagnosis**	

	Eclampsia, uterine rupture, ectopic pregnancy

^a ^free flow infusion refers to a massive infusion of fluids in case of shock

Data were collected retrospectively between October 2004 and October 2005, covering all admissions between November 1^st ^2003 and October 31^st ^2004. Data on complications, mode of delivery, age, parity and birth outcome were extracted from registers in the delivery ward, surgical ward, obstetric ward, and intensive care unit, linking women by hospital admission number. All women reported to have any complication, surgery or a perinatal death in any of the registers were selected for detailed case note review. For maternal deaths, further efforts were made to screen all registers from other female wards in the hospital. The data collection was done by eight medical doctors using structured extraction forms. They scrutinized the case notes and reported the diagnoses listed by the providers, the presence of specific near miss criteria, and the timing of the occurrence of complications. They also differentiated cases admitted in a critical state from those whose life-threatening complication developed during hospitalization, relying on the information on vital signs and conditions at admission, as well as on the data collectors' (medical doctor) judgment in the case of missing information.

The statistical analysis was descriptive; proportions were compared using the Chi Square.

Ethical approval by an ethic committee of the University of Indonesia was obtained prior to field work. In addition, written consent for collecting and sharing the data was obtained from the head of each hospital, and the obstetricians gave verbal approval. The data were stored electronically as part of the Immpact data-base in Indonesia and UK offices.

## Results

There were 5,669 pregnancy-related admissions in one year in the four hospitals: 2,803 in Serang hospital, 1,212 in Pandeglang hospital, 873 in Budi Asih and 781 in Kencana. Information from the registers identified 4,571 (80.6%) women as having had a complication, and case notes were found in 4,270 (93.4%) of those.

**Figure 1:**
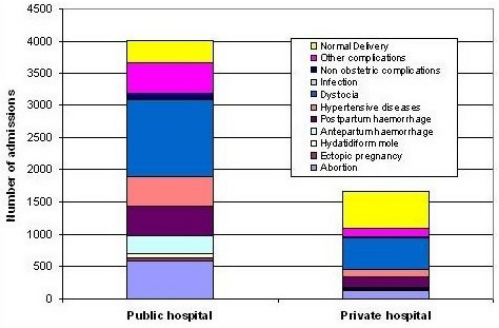
Patterns of obstetric admissions in two public and two private hospitals in Serang and Pandeglang (November 2003-October 2004).

Figure 1 shows the main reasons for pregnancy-related admissions in public and private hospitals. About a third of admissions were for dystocia, both in public (29.7%) and private (29.9%) hospitals. This was followed by early pregnancy loss in public hospitals (17.6%) and postpartum hemorrhage in private hospitals (10.1%). Abortions represented 14.7% and 7.9% of all admissions in public and private hospitals respectively. Private hospitals admitted more women with normal delivery or non maternal complications such as fetal distress or cord prolapsed than public hospitals (41.6% versus 20.5%).

There were 763 cases of near miss in all four hospitals. The near miss criteria and their frequency are shown in Table 1. Women could fall under one or more criteria for organ dysfunction regardless of whether they had any of the three clinical diagnoses or any of the five management criteria. The majority (77.3%) of near miss had one major organ dysfunction, 16.0% had two, 4.6% had three, and 2.0% had four or more.

Vascular dysfunction was by far the most common organ dysfunction (77.7%) followed by cardiac (5.1%) and renal dysfunction (4.5%) (Table 2). Septic shock was only found in one record. Vascular dysfunction was largely defined by transfusion of two or more units of blood, hypovolaemic shock or massive hemorrhage as recorded in the notes. Making the criteria more stringent by increasing the need for blood transfusion to three or more and four or more units reduced the total number of near miss to 709 (92.9%) and 679 (89.0%) cases respectively (data not shown). Similarly, removing recorded massive hemorrhage as a criterion slightly reduced the number of near miss to 754. Finally, excluding management and clinical criteria reduces the number of near-miss from 763 to 640, with the most important changes occurring for ectopic pregnancy (from 67 to 39) and eclampsia (from 97 to 41).

**Table 2:** Type and frequency of near-miss criteria in 763 near miss cases admitted to four hospitals in Serang and Pandeglang (November 2003 – October 2004)

***ORGAN DYSFUNCTION***	**N (%)**
**Any cardiac dysfunction**	**39 (5.1)**
Pulmonary oedema	7 (0.9)
Cardiac arrest	7 (0.9)
Cardiac failure	27 (3.5)
**Pulmonary embolism**	**2 (0.3)**
**Any vascular dysfunction**	**593 (77.7)**
Hypovolemia requiring 2 or more units of blood	485 (63.6)
Blood loss with hypovolemic shock	227 (29.8)
Infusion and/or transfusion of > = 1 litre per 2 hours	38 (5.0)
Free flow infusion	278 (36.4)
Massive haemorrhage recorded in notes	221 (29.0)
**Immunological dysfunction**	**1 (0.1)**
Septic shock	1 (0.1)
**Respiratory dysfunction**	**1 (0.1)**
Intubation or ventilation for reasons other than general anaesthesia	1 (0.1)
Oxygen saturation on pulse oximetry < 90% leading to ventilation	1 (0.1)
**Renal dysfunction**	**34 (4.5)**
Oliguria < 30 ml per hour or < 400 ml per 24 hours	34 (4.5)
Shock not responsive to intravascular rehydration or diuresis	1 (0.1)
Creatinine clearance test	2 (0.3)
Haemodialysis	2 (0.3)
**Liver dysfunction**	**19 (2.5)**
Jaundice in pre eclampsia	1 (0.1)
Abnormal liver function tests	18 (2.4)
**Coagulation dysfunction**	**8 (1.0)**
Acute thrombocytopenia	3 (0.4)
Prolonged bleeding time	6 (0.8)
Abnormal APTT or TT	1 (0.1)
Coagulopathy	2 (0.3)
**Any Cerebral dysfunction**	**14 (1.8)**
Coma	9 (1.2)
Cerebral oedema	5 (0.7)
Seizures other than eclampsia	2 (0.3)
**MANAGEMENT BASED CRITERION**	**97 (12.7)**
Intensive care admission	38 (5.0)
Emergency hysterectomy	19 (2.5)
Needs resuscitation	14 (1.8)
Anaesthetic accident	1 (0.1)
Referral to tertiary hospital	35 (4.6)
**CLINICAL DIAGNOSIS**	**193 (25.3)**
Eclampsia	99 (13.0)
Uterine rupture	26 (3.4%)
Ectopic pregnancy	68 (8.9%)

Note: numbers add up to > 100% because one near miss can have more than one criterion

Table 3 shows the distribution of admissions, near miss and maternal deaths in public and private hospitals. The proportion of near miss was much greater in public than in private hospitals (17.3% versus 4.2%, p = 0.000). Similarly, only one maternal death was reported in the private hospitals (0.1% of all admissions), compared to 63 (1.6% of all admissions) in the public hospitals. Near miss cases were extremely common among admissions for ante partum and postpartum hemorrhage (Table 3). In public hospitals, 41.2% of admissions for postpartum hemorrhage, 40.6% for ante partum hemorrhage, and 32.3% for hypertensive diseases were classified as near miss. Women admitted with abortions also had their fair share of near miss (16.3%). Maternal mortality, on the other hand was highest among non-obstetric admissions (13.5%) followed by hypertensive diseases (4.7%), ante partum (2.5%) and postpartum hemorrhage (2.0%).

**Table 3:** Admissions, near miss and deaths according to the main diagnosis during hospitalisation in four hospitals in Serang and Pandeglang district (November 2003-October 2004)

	**DISTRICT hospitalS**			**Private hospitals**		
	**All**	**Near miss n (%)**	**Deaths n (%)**	**All**	**Near miss n (%)**	**Deaths n (%)**

**Early pregnancy loss**	**706**	**164 (23.2)**	**3 (0.4)**	**154**	**22 (14.6)**	**-**
Abortion	589	96 (16.3)	1 (0.2)	131	3 (2.3)	-
Ectopic pregnancy	49	48 (98.0)	1 (2.0)	19	19 (100.0)	-
Hydatidiform mole	68	20 (29.4)	1 (1.5)	4	-	-
**Antepartum haemorrhage**	**276**	**112 (40.6)**	**7 (2.5)**	**26**	**6 (23.1)**	**-**
Placenta praevia	185	75 (40.5)	3 (1.6)	19	5 (26.3)	-
Abruptio placentae	45	27 (60.0)	2 (4.4)	2	-	-
Unspecified	46	10 (21.7)	2 (4.3)	5	1 (20.0)	-
**Postpartum haemorrhage**	**442**	**182 (41.2)**	**9 (2.0)**	**168**	**10 (6.0)**	**-**
Uterine atony	24	17 (70.8)	-	1	1 (100.0)	-
Retained placenta	117	56 (47.9)	4 (3.4)	6	1 (16.7)	-
Tear	163	20 (12.3)	1 (0.6)	147	-	-
Unspecified	138	89 (64.5)	4 (2.9)	14	8 (57.1)	-
**Hypertensive diseases**	**468**	**151 (32.3)**	**22 (4.7)**	**95**	**12 (12.6)**	**-**
Pre-eclampsia	362	60 (16.6)	7 (1.9)	89	6 (6.7)	-
Eclampsia	106	91 (85.8)	15 (14.2)	6	6 (100.0)	-
**Dystocia**	**1194**	**72 (6.0)**	**10 (0.8)**	**494**	**17 (3.4)**	**1 (0.2)**
Uterine rupture	30	24 (80.0)	6 (20.0)	3	2 (66.7)	1 (33.3)
Bandl's ring	19	5 (26.3)	-	8	-	-
CPD and prolonged labour	855	34 (4.0)	2 (0.2)	371	13 (3.5)	-
Malpresentation	290	9 (3.1)	2 (0.7)	112	2 (1.8)	-
**Infection**	**17**	**1 (5.9)**	**-**	**0**	**-**	**-**
**Non obstetric complications**	**89**	**12 (13.5)**	**12 (13.5)**	**29**	**2 (6.9)**	**-**
Other complications*	**472**	**-**	**-**	**126**	**-**	**-**
**Normal Delivery**	**351**	**-**	**-**	**562**	**-**	**-**
**All**	**4015**	**694 (17.3)**	**63 (1.6)**	**1654**	**69 (4.2)**	**1 (0.1)**

*Other complications include premature rupture of membranes anaemia, fetal distress, post-term, cord strangulation, twins, and hyperemesis gravidarum

Among all life-threatening complications (i.e. near miss and death), mortality was highest for non-obstetric admissions (50%), followed by hypertensive diseases (17.8%) and dystocia (14.5%) (Table 4). Among life-threatening non-obstetric admissions, heart failure was the most common diagnosis (2 deaths and 6 near miss), followed by tuberculosis (2 deaths and 2 near miss), diabetes (1 death), paralytic ileums (1 death), sepsis (1 death), anemia (1 near miss), breast cancer with metastasis to the liver (1 near miss), chronic renal failure (1 death), dengue haemorrhagic fever (1 death and 1 near miss), and asthma (2 deaths).

**Table 4:** Life threatening (near miss and maternal death) admissions according to the main diagnosis during hospitalisation in four hospitals Serang and Pandeglang districts (November 2003-October 2004)

	**DISTRICT hospitalS**			**Private hospitals**			**All hospitals**		
	**Near miss and death**	**Near miss n (%)**	**Deaths n (%)**	**Near miss and death**	**Near miss n (%)**	**Deaths n (%)**	**Near miss and death**	**Near miss n (%)**	**Deaths n (%)**

**Early pregnancy loss**	**167**	**164 (98.2)**	**3 (1.8)**	**22**	**22 (100.0)**	**-**	**189**	**186 (98.4)**	**3 (1.6)**
Abortion	97	96 (99.0)	1 (1.0)	3	3 (100.0)	-	100	99 (99.0)	1 (1.0)
Ectopic pregnancy	49	48 (98.0)	1 (2.0)	19	19 (100.0)	-	68	67 (98.5)	1 (1.5)
Hydatidiform mole	21	20 (95.2)	1 (4.8)	-	-	-	21	20 (95.2)	1 (4.8)
**Antepartum haemorrhage**	**119**	**112 (94.1)**	**7 (5.9)**	**6**	**6 (100.0)**	**-**	**125**	**118 (94.4)**	**7 (5.6)**
Placenta praevia	78	75 (96.2)	3 (3.8)	5	5 (100.0)	-	83	80 (96.4)	3 (3.6)
Abruptio placentae	29	27 (93.1)	2 (6.9)	-	-	-	29	27 (93.1)	2 (6.9)
Unspecified	12	10 (83.3)	2 (16.7)	1	1 (100.0)	-	13	11 (84.6)	2 (15.4)
**Postpartum haemorrhage**	**191**	**182 (95.3)**	**9 (4.7)**	**10**	**10 (100.0)**	**-**	**201**	**192 (95.5)**	**9 (4.5)**
Uterine atony	17	17 (100.0)	-	1	1 (100.0)	-	18	18 (100.0)	-
Retained placenta	60	56 (93.3)	4 (6.7)	1	1 (100.0)	-	61	57 (93.4)	4 (6.6)
Tear	21	20 (95.2)	1 (4.8)	-	-	-	21	20 (95.2)	1 (4.8)
Unspecified	93	89 (95.7)	4 (4.3)	8	8 (100.0)	-	101	97 (96.0)	4 (4.0)
**Hypertensive diseases**	**173**	**151 (87.3)**	**22(12.7)**	**12**	**12 (100.0)**	**-**	**185**	**163 (88.1)**	**22 (11.9)**
Pre-eclampsia	67	60 (89.6)	7 (10.4)	6	6 (100.0)	-	73	66 (90.4)	7 (9.6)
Eclampsia	106	91 (85.8)	15 (14.2)	6	6 (100.0)	-	112	97 (86.6)	15 (13.4)
**Dystocia**	**82**	**72 (87.8)**	**10 (12.2)**	**18**	**17 (94.4)**	**1 (5.6)**	**100**	**89 (89.0)**	**11 (11.0)**
Uterine rupture	30	24 (80.0)	6 (20.0)	3	2 (66.7)	1 (33.3)	33	26 (78.8)	7 (21.2)
Bandl's ring	5	5 (100.0)	-	-	-	-	5	5 (100.0)	-
CPD and prolonged labour	36	34 (94.4)	2 (5.6)	13	13 (100.0)	-	49	47 (95.9)	2 (4.1)
Malpresentation	11	9 (81.8)	2 (18.2)	2	2 (100.0)	-	13	11 (84.6)	2 (15.4)
**Infection**	**1**	**1 (100.0)**	**-**	**-**	**-**	**-**	**1**	**1 (100.0)**	**-**
**Non obstetric complications**	**24**	**12 (50.0)**	**12 (50.0)**	**2**	**2 (100.0)**	**-**	**26**	**14 (53.8)**	**12 (46.2)**
Other complications*	**-**	**-**	**-**	**-**	**-**	**-**	**-**	**-**	**-**
**Normal Delivery**	**-**	**-**	**-**	**-**	**-**	**-**	**-**	**-**	**-**
**All**	**757**	**694 (91.7)**	**63 (8.3)**	**70**	**69 (98.6)**	**1 (1.4)**	**827**	**763 (92.3)**	**64 (7.7)**

*Other complications include premature rupture of membranes anaemia, fetal distress, post-term, cord strangulation, twins, and hyperemesis gravidarum

Severe anemia was extremely common among near-miss (Table 5). More than half (56.4%) of near-miss cases had hemoglobin levels below 8 g/dl. Severe anemia was less common among maternal deaths (21.9%), though hemoglobin had not been recorded in 42.2% of the women who died. Three quarters (77.6%) of near-miss cases with hemoglobin levels below 8 g/dl were associated with blood loss because of early pregnancy loss, or ante or postpartum hemorrhage (data not shown).

**Table 5:** Level of haemoglobin (Hb) among admissions, near miss and deaths in four hospitals in Serang and Pandeglang (November 2003 – October 2004)

	**Near miss n (%)**	**Death n (%)**	**Other n (%)**	**All n (%)**
**Hb < 6 g/dl**	198 (26.0)	8 (12.5)	45 (0.9)	251 (4.4)
**Hb 6 – 7.9 g/dl**	232 (30.4)	6 (9.4)	236 (4.9)	474 (8.4)
**Hb 8 – 9.9 g/dl**	138 (18.1)	10 (15.6)	732 (15.1)	880 (15.5)
**Hb > = 10 g/dl**	93 (12.2)	13 (20.3)	1254 (25.9)	1360 (24.0)
**No Hb, with clinical anaemia**	17 (2.2)	3 (4.7)	13 (0.3)	33 (0.6)
**No Hb, with no recorded clinical anaemia**	85 (11.1)	24 (37.5)	2562 (52.9)	2671 (47.1)
**All**	763 (100.0)	64 (100.0)	4842 (100.0)	5669 (100.0)

**Figure 2:**
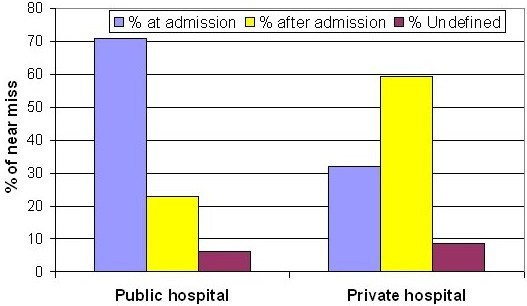
Near miss at and after admission in two public and two private hospitals in Serang and Pandeglang (November 2003-October 2004).

The majority (67.2%) of near miss were in a critical state at admission and this proportion was higher in public than in private hospitals (70.7% and 31.9% respectively, p = 0.000) (Figure 2). Interestingly, about a third (31.0%) of the 577 near miss who had given birth were admitted postpartum, compared to only 5.8% of all other birth related admissions (p = 0.000). Similar proportions were found among the 61 birth-related maternal deaths, 29.5% of whom were admitted in the postpartum (data not shown).

## Discussion

This is the first hospital-based study to document near miss in public and private hospitals in Indonesia. In these hospitals where most women admitted had an obstetric complication the main causes of near miss were hemorrhage and hypertension, reflecting the main causes of maternal death in Indonesia. Close to a fifth of admissions in the two public hospitals were associated with near miss, though near misses were much less common in private hospitals. The majority of near miss in public hospitals arrived in the hospitals in a state of emergency, strongly suggesting delays in reaching the hospitals.

There is no consensus on how to define near miss [1], and definitions have evolved from general clinical concepts of obstetric morbidity [7] to those of organ dysfunction [6]. Agreeing on standard criteria of near miss was not straightforward, and vascular dysfunction in particular was difficult to characterize. Vascular dysfunction is often defined using criteria for blood transfusion, generally using four or five units as the cut off for near miss [2,6,8,9] . Due to the scarcity of blood products in the two public hospitals, the number of transfused units in our study was set at two units. Making the criteria more stringent by increasing the required number of units to three or four reduced the number of near miss by about ten percent, though hemorrhage remained the leading cause. The extremely low levels of hemoglobin among near miss also suggest that the thresholds may have been sufficiently extreme to identify severe cases.

The reliance on management criteria to define near miss will continue to pose problems when the aim is to compare data across hospitals. Admission to intensive care, for example, continues to be a commonly used criterion, even in Western countries [1,2,6,8]. In Scotland, only a third (28%) of all near miss cases were admitted to intensive care; in our main district hospital in Serang this proportion was only 4.1%. Admission criteria to intensive care vary between countries, hospitals and clinicians and the capacity and location of the intensive care unit also influences the number of admissions [10]. The reliance on clinical diagnoses also introduces variability. Not all women with eclampsia nearly die, not all women with an ectopic pregnancy are critically ill and a scar dehiscence in a woman with a previous caesarean section may not be a uterine rupture. A definition using only organ system dysfunction would be more reproducible across countries and between institutions, but this requires the availability of good clinical and laboratory records, which may not be available everywhere.

The retrospective nature of the data collection may have introduced some bias. Case notes were found for a relatively large proportion of cases (75.4%, data not shown), but some case notes were clearly incomplete. The extremely low rates of immunological and respiratory dysfunction, for example, may reflect the poor recording of such complications, and the incidence of these complications in the hospitals may have been underestimated. The assessment of whether the woman was in a critical state at or after admission may also have been erroneous in some cases, and these results have to be interpreted with caution

The main causes of near miss identified here, such as hemorrhage and hypertensive diseases, are similar to those found elsewhere [2,6,11]. Sepsis, on the other hand, was very rare, confirming findings from another study in Indonesian hospitals [12]. A notable finding is the high number of near miss following abortion. Induced abortion is illegal in Indonesia, and the case notes did not distinguish spontaneous from induced abortion. Monitoring abortion-related hospital admissions has been suggested as a useful way to quantify the magnitude of the adverse health effects of unsafe abortion in developing countries [13]. Measuring trends in the severity of cases through ascertainment of near miss may be a promising indicator of the burden of unsafe abortion.

Women with obstetric complications, near miss and maternal deaths were all less common in the private than in the public sector. The two private hospitals accounted for about a third of all admissions, yet they only represented 9.0% and 1.6% of near miss and maternal deaths respectively. It is clear that healthier, and most likely wealthier, women selectively opt for obstetric care in the private sector and the differences in morbidity and mortality between public and private hospitals are most certainly due to case-mix differences. Private hospitals are also likely to refer women with severe complications to public hospitals, thereby keeping the incidence of near miss lower. These differences do not imply that care is less good in one sector than another.

Quantifying the magnitude, causes and timing of near miss in hospitals is only a first step in the investigation of near miss. The large number of near miss upon arrival begs the question as to why women get to hospital so late. Births in hospital are very infrequent in Indonesia, and caesarean section rates are extremely low among large sections of the population [14]. Interviewing near miss may help to elucidate the barriers to hospital care. Even if women arrive late, however, much can be done to save their and their babies' life, and a review into the care received in the hospital – both in the public and private sector – may lead to positive changes in the procedures and resources available for the management of obstetric complications [15].

## Conclusion

This is the first study to document near miss in public and private hospitals in Indonesia. Close to a fifth of admissions in public hospitals were associated with near miss; and the critical state in which the women arrived suggest important delays in reaching the hospitals. Even though the private sector takes an increasingly larger share of facility-based births in Indonesia, managing obstetric emergencies remains the domain of the public sector.

## Competing interests

The author(s) declare that they have no competing interests.

## Authors' contributions

CR has made substantial contributions to conception and design of the study, interpretation of data, drafted the manuscript and revising it critically for important intellectual content. CS has been involved in drafting the manuscript and revising it critically for important intellectual content. FN participated in data acquisition and interpretation. PD participated in data acquisition, statistical analysis and versions of the manuscript. AA has been involved in the design of the study, acquisition of data and interpretation of data, and in drafting the manuscript. The manuscript has been read and approved by all authors.

## Pre-publication history

The pre-publication history for this paper can be accessed here:


